# Sequencing and functional annotation of the whole genome of the filamentous fungus *Aspergillus westerdijkiae*

**DOI:** 10.1186/s12864-016-2974-x

**Published:** 2016-08-15

**Authors:** Xiaolong Han, Alolika Chakrabortti, Jindong Zhu, Zhao-Xun Liang, Jinming Li

**Affiliations:** 1 State Key Laboratory of Organ Failure Research, Division of Nephrology, Nanfang Hospital, Southern Medical University, Guangzhou, Guangdong Province People’s Republic of China; 2 Department of Bioinformatics, School of Basic Medical Sciences, Southern Medical University, Guangzhou, Guangdong Province People’s Republic of China; 3 School of Biological Sciences, Nanyang Technological University, 60 Nanyang Drive, Singapore, Republic of Singapore

**Keywords:** Fungi, *Aspergillus westerdijkiae*, Comparative genomics, Secondary metabolite, Orthologues, Phylogeny

## Abstract

**Background:**

*Aspergillus westerdijkiae* produces ochratoxin A (OTA) in *Aspergillus* section *Circumdati*. It is responsible for the contamination of agricultural crops, fruits, and food commodities, as its secondary metabolite OTA poses a potential threat to animals and humans. As a member of the filamentous fungi family, its capacity for enzymatic catalysis and secondary metabolite production is valuable in industrial production and medicine. To understand the genetic factors underlying its pathogenicity, enzymatic degradation, and secondary metabolism, we analysed the whole genome of *A. westerdijkiae* and compared it with eight other sequenced *Aspergillus* species.

**Results:**

We sequenced the complete genome of *A. westerdijkiae* and assembled approximately 36 Mb of its genomic DNA, in which we identified 10,861 putative protein-coding genes. We constructed a phylogenetic tree of *A. westerdijkiae* and eight other sequenced *Aspergillus* species and found that the sister group of *A. westerdijkiae* was the *A. oryzae* - *A. flavus* clade. By searching the associated databases, we identified 716 cytochrome P450 enzymes, 633 carbohydrate-active enzymes, and 377 proteases. By combining comparative analysis with Kyoto Encyclopaedia of Genes and Genomes (KEGG), Conserved Domains Database (CDD), and Pfam annotations, we predicted 228 potential carbohydrate-active enzymes related to plant polysaccharide degradation (PPD). We found a large number of secondary biosynthetic gene clusters, which suggested that *A. westerdijkiae* had a remarkable capacity to produce secondary metabolites. Furthermore, we obtained two more reliable and integrated gene sequences containing the reported portions of OTA biosynthesis and identified their respective secondary metabolite clusters. We also systematically annotated these two hybrid *t1pks-nrps* gene clusters involved in OTA biosynthesis. These two clusters were separate in the genome, and one of them encoded a couple of GH3 and AA3 enzyme genes involved in sucrose and glucose metabolism.

**Conclusions:**

The genomic information obtained in this study is valuable for understanding the life cycle and pathogenicity of *A. westerdijkiae*. We identified numerous enzyme genes that are potentially involved in host invasion and pathogenicity, and we provided a preliminary prediction for each putative secondary metabolite (SM) gene cluster. In particular, for the OTA-related SM gene clusters, we delivered their components with domain and pathway annotations. This study sets the stage for experimental verification of the biosynthetic and regulatory mechanisms of OTA and for the discovery of new secondary metabolites.

**Electronic supplementary material:**

The online version of this article (doi:10.1186/s12864-016-2974-x) contains supplementary material, which is available to authorized users.

## Background

*Aspergillus westerdijkiae* (CBS 112803 = NRRL 3174), a filamentous fungus branched from the *A. ochraceus* taxon [1], has a worldwide distribution and mainly colonizes agricultural crops and various food commodities, such as coffee, beer, wine, milk, grapes, oranges, and juice [2–4]. Previous studies have shown that *A. westerdijkiae* is also present in house dust and indoor air fallout [5], and some other subtypes are found in deep sea environments [6, 7].

Filamentous fungi of the *Aspergillus* genus are among the most prolific sources of secondary metabolites with biomedical and commercial importance [6]. *A. westerdijkiae* is known as the main ochratoxin A (OTA)-producing species, of which approximately 70 % of the strains are able to produce OTA [8]. OTA, a polyketide secondary metabolite, is potentially carcinogenic in humans through its induction of oxidative DNA damage [9] and is neurotoxic, with a strong affinity for the brain [10]. In addition, OTA can induce renal adenomas and hepatocellular carcinomas in rodents [11]. Numerous studies aimed at the mechanism of OTA production and its activity have been conducted [12–16]. Researchers also developed a real-time quantitative PCR protocol to detect and quantify *A. westerdijkiae* contamination in grapes and green coffee beans, focussing on the ITS1-5.8S-ITS2 region within the rDNA unit, which serves as a tag to evaluate *A. westerdijkiae* contamination and has been frequently used to discriminate at the species level [17].

Thus far, phylogenetic studies examining *A. westerdijkiae* have had been performed with only three or four gene loci [1, 18]. A phylogenetic analysis using a small number of concatenated genes may have a high probability for supporting conflicting topologies, while an analysis with whole-genome data could provide greater resolving power by allowing trees to be constructed based on all available concatenated sets of genes [19]. In this study, we used 561 highly conserved single-copy orthologous gene sets found in whole genome-wide searches to infer the phylogenetic relationships between *A. westerdijkiae* and the eight other *Aspergillus* species.

Enzymatic degradation of plant polysaccharides in fungi is notable for its relevance in many industrial applications, such as paper, food, animal feed, biofuel, and chemicals [20–22]. Fungi have been used to subsist on various types of plant biomass as a carbon source by producing enzymes that degrade cell well polysaccharides in the exterior milieu into simple monomers for nutrition [21]. Localized degradation of the cell wall also allows for penetration and spreading across host tissues [23]. The CAZy database (http://www.cazy.org) has classified the enzymes degrading or modifying plant polysaccharides into carbohydrate-active enzymes and has divided them into different families. A previous study comparing eight sequenced *Aspergilli* genomes revealed that the related fungi employed diverse enzymatic strategies to degrade plant biomass and provided detailed categorization for these species. This study provided practical knowledge to further analyse the capability of plant polysaccharide depolymerization of *A. westerdijkiae* [22]. Interestingly, the latest studies revealed that *A. westerdijkiae* OTA production had no positive relationship with growth or sporulation and was markedly variable both qualitatively and quantitatively among different substrates [24, 25].

Fungi are also deemed to be a potential source of proteases due to their broad biochemical diversity [26]. Enzymatic proteolysis has many extremely important applications in the pharmaceutical, medical, food, and biotechnological industries [27]. The MEROPS database (http://merops.sanger.ac.uk) is an integrated resource for proteases and the proteins that inhibit proteases. This database has organized peptidases into various families on the basis of statistically significant similarities in amino acid sequences and includes a batch Blast prediction tool [28].

Until now, studies examining *A. westerdijkiae* have only employed low-throughput experimental approaches or *in vitro* observation to check for known characteristics and to explore unknown features. These methods are useful for making reliable conclusions but are not ideal for exploring unknown characteristics.

In this study, we sequenced and assembled a complete genome of *A. westerdijkiae* NRRL 3174 using an Illumina MiSeq platform. We analysed the genome to identify the genes that might be secreted and might contribute to pathogenicity and secondary metabolite biosynthesis. Domains of each component of all of the predicted SM gene clusters were annotated, and we provided the detailed annotation for two putative OTA-related gene clusters, a putative Notoamide biosynthetic gene cluster and a putative Hexadehydro-astechrome (HAS) biosynthetic gene cluster. We also examined the classification of the plant polysaccharide degradation enzymes and found that the union of GH3 and AA3 present in one of the OTA-related SM gene clusters might be associated with the responses of *A. westerdijkiae* growing in different media. We also compared its genome and proteome similarities, evolutionary relationship, and plant biomass degradation potential to those of eight sequenced *Aspergillus* species: *A. flavus*, *A. clavatus*, *A. fumigatus*, *A. nidulans*, *A. niger*, *A. oryzae*, *A. terreus*, and *N. fischeri* (Additional file 1: Table S1). The information contained in this study could be helpful for understanding the molecular mechanisms and the evolution of this important *Aspergillus* species.

## Results and discussion

### Genome details and comparative analysis

The *A. westerdijkiae* genome was sequenced to 142.0x coverage using an Illumina MiSeq platform [29]. The complete sequences for the Whole Genome Shotgun projects have been deposited at DDBJ/EMBL/GenBank under the accession numbers LKBE00000000. The version described in this paper is the first version LKBE00000000.1. The genome assembly is approximately 36 M in size and includes 239 scaffolds with an N50 length of 1,603,627 bp (Table 1). Comparatively, *A. westerdijkiae* has a similar genome size to the sister group *A. flavus* and *A. oryzae*, which differ considerably from the others (Table 2). To assess the gene space in the *A. westerdijkiae* genome, we used a set of 248 core eukaryotic genes to perform CEGMA prediction, which showed that 234 out of 248 (94.35 %) genes were completely matched to our assembled genome. This finding suggested that we had successfully assembled approximately 95 % of all *A. westerdijkiae* genes [30]. In this case, 10,861 protein-coding genes were predicted using the annotation pipeline MAKER2 to integrate evidence from multiple databases with the gene-finding programs SNAP and AUGUSTUS. The predicted transcripts and proteins have been deposited in supplementary information (Additional file 2). These putative protein-coding genes cover 30.68 % of the nucleotide sequence of the *A. westerdijkiae* genome. Compared to increasing the genome size, using a strict prediction method may lead to a relatively lower value of gene density in *A. westerdijkiae* (Table 2). Of the 10,861 predicted genes of *A. westerdijkiae*, 3964 (36.5 %) were assigned as hypothetical, and 458 (4.2 %) were considered as unique, without any matches in the NCBI non-redundant (nr) database and the UniProt knowledgebase.

**Table 1:** Genome characteristics and predicted features of the assembled A. westerdijkiae strain CBS 112803

Genome	Value
Assembly size (Mb)	36.1
G + C content (%)	50.2
N50 length (bp)	1,603,627
N50 (num of scaffolds)	8
N90 (num of scaffolds)	22
Average length (bp)	113,426
Assembled contigs	3022
Assembled scaffolds	239
Assembly gap length	1,575,768
Predicted proteins	10,861
Predicted proteins (>100 amino acids)	10,530

**Table 2:** Summary of several main features for *A. westerdijkiae* and eight sequenced Aspergillus genomes

Species	Genome size (Mb)	Chrs	%GC	Proteins	Density (%)	tRNAs	Ref.
A. westerdijkiae	36.1	-	50.2	10,861	30.68	208	-
A. clavatus	27.7	8	49.2	9120	32.92	269	[33]
A. fumigatus	29.4	8	49.9	9887	33.63	179	[58]
A. flavus	36.8	8	48.35	12,587	34.20	248	[60]
A. nidulans	30.1	8	50.32	10,560	35.08	188	[34]
A. niger	33.9	8	50.36	8592	25.35	277	[59]
A. oryzae	36.7	8	48.24	12,063	32.87	270	[31]
A. terreus	29.0	8	52.90	10,406	35.88	150	[33]
N. fischeri	32.0	8	49.43	10,403	32.51	374	[33]

**Fig. 1:**
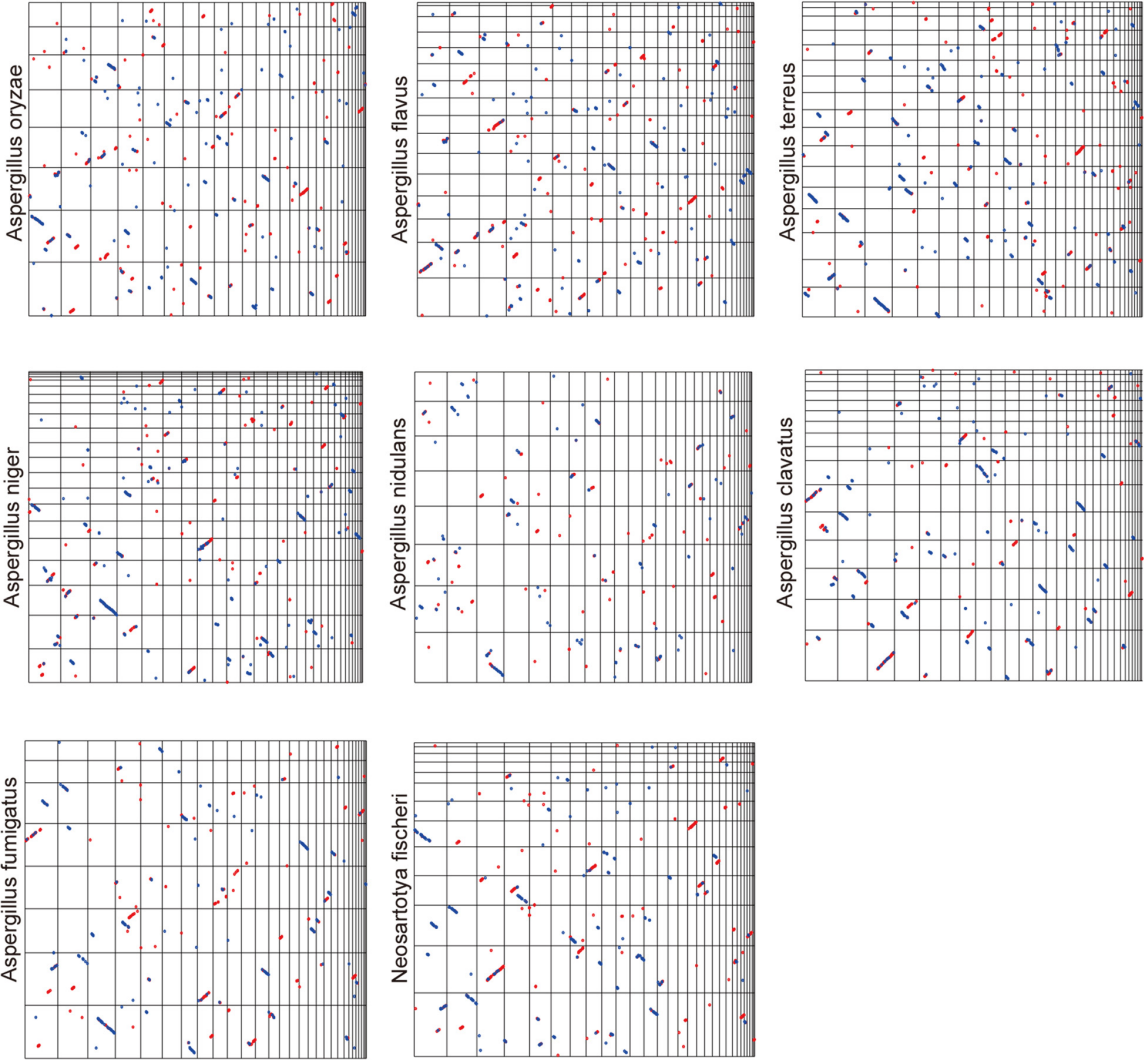
Dotplot view of the Nucmer genome alignment between the *A. westerdijkiae* genome and the other selected *Aspergillus* genomes (*A. oryzae, A. flavus, A. terreus, A. niger, A. fumigatus, N. fischeri, A. clavatus, A. nidulans*). Each subgraph represents a comparison between the *A. westerdijkiae* genome and another selected *Aspergillus* genome. In each subgraph, the *x*-axis refers to the 29 largest supercontigs of *A. westerdijkiae* sorted in decreasing order of size, and the *y*-axis refers to the supercontigs of another selected genome sorted in decreasing order of size

Over 200 scaffolds of *A. westerdijkiae* were finally assembled in this study. Any gaps between the scaffolds might be due to one of the reasons. Comparative analysis could be used to find conserved regions among the genomes and to infer the probable collinearity of the assembled scaffolds. Pairwise comparisons between *A. westerdijkiae* and the eight sequenced *Aspergillus* genomes were conducted by applying the scaffolds greater than 100 kb in these genomes to the NUCmer programs (Fig. 1) [31]. A total of 29 scaffolds from *A. westerdijkiae* larger than 100 kb in length and covering more than 97 % of the genome were extracted (Additional file 1: Table S2). Scaffolds 9, 17, and 75 displayed relative conservation among these sequenced *Aspergilli*, and scaffolds 5 and 23 were shown to be possibly syntenous.

Although in the same genus, the *Aspergilli* also show extensive structural reorganization and differ in their genome sequences. Only less than 0.2 % of the nucleotides of *A. westerdijkiae* were shared with *Aspergilli*, with an average of 88 % identity. And the alignment displayed an average of 68 % amino acid identity, comparable to similar findings among *A. fumigatus*, *A. oryzae*, and *A. nidulans* (Additional file 3: Table S3) [32]. *A. westerdijkiae* and *A. nidulans* showed the lowest similarity; therefore, we selected *A. nidulans* as the outgroup for the phylogenetic tree construction.

### Phylogenetic relationship

**Fig. 2:**
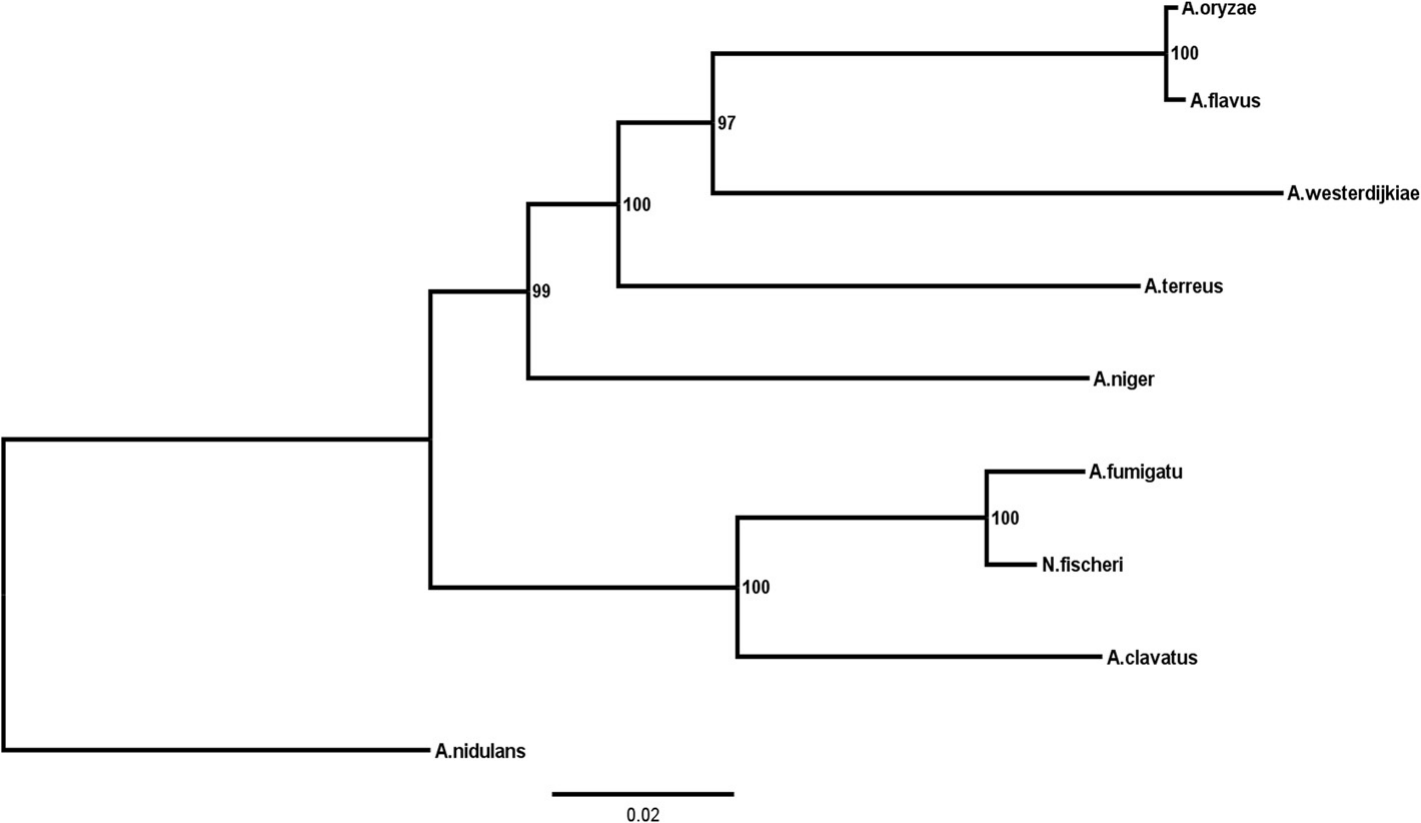
A maximum likelihood phylogenomic tree was inferred on the basis of 561 concatenated orthologous single-copy amino acid sequences of the nine *Aspergillus* genomes (*A. oryzae, A. flavus, A. westerdijkiae, A. terreus, A. niger, A. fumigatus, N. fischeri, A. clavatus, A. nidulans*) using the Dayhoff model in TREE-PUZZLE

In this study, we used the entire dataset of concatenated single-copy orthologous proteins to build the phylogenetic tree. Using the Perl script Proteinortho, 561 orthologous clusters were present as single copies across the nine *Aspergillus* species. We further built the maximum likelihood (ML) tree based on the orthologous proteins, selecting *A. nidulans* as the outgroup (Fig. 2) [33]. This tree illustrated that *A. westerdijkiae* was most closely related to *A. oryzae* and *A. flavus* and the opportunistic pathogen *A. terreus. A. oryzae* has been used in the fermentation process of several traditional Japanese beverages and sauces [34]. *A. flavus* is well known for producing the potent carcinogen aflatoxin [35]. The profile of this phylogenetic tree was consistent with those reported in previous studies [32, 36].

### Predicted secreted proteins involved in virulence or detoxification

**Fig. 3:**
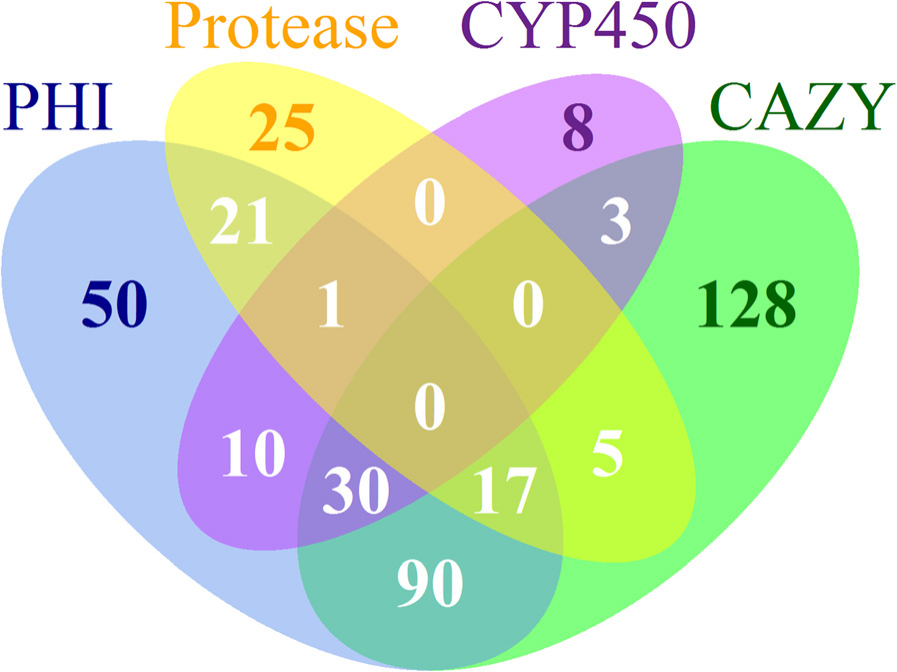
Venn-plot showing the intersections among the secreted PHI proteins (blue), secreted proteases (yellow), secreted CYP450 enzymes (darkorchid), and secreted CAZymes (green)

*A. westerdijkiae* is one of the phytopathogenic fungi that can cause serious diseases. Secreted enzymes play crucial roles in pathogenicity and virulence [37]. With the *in silico* pipeline described in the Methods section, 801 out of the 10,861 (7.38 %) predicted proteins were potentially secreted proteins, close to the average 8 % found in other *Aspergillus* species [38]. By performing a whole-proteome Blast against the pathogen-host interaction (PHI) database, 3124 out of 10,861 (28.76 %) predicted proteins encoded by the *A. westerdijkiae* genome were determined to share homology with the genes implicated in pathogenicity and virulence in the PHI-Base, of which 219 (0.7 %) putative PHI-related proteins are potential secreted proteins. Cytochrome P450 enzymes not only participate in the production of the metabolites important for the organism’s internal needs but also play critical roles in adaptation to diverse environments by modifying harmful environmental chemicals. Using Blast to search the fungal Cytochrome P450 database, we found that 716 out of 10,861 (6.6 %) predicted proteins matched to 7711 out of 9209 (83.73 %) CYP450s, of which 52 putative CYP450 enzymes were predicted to be secreted, most of which were involved in pathogen-host interactions (Fig. 3, Additional file 4: Table S4). According to the result of orthologous analysis, 38 putative PHI-related genes can be found across all the sequenced *Aspergilli*, while 31 genes were only expressed in *A. westerdijkiae*.

### Carbohydrate-active enzymes (CAZymes)

**Fig. 4:**
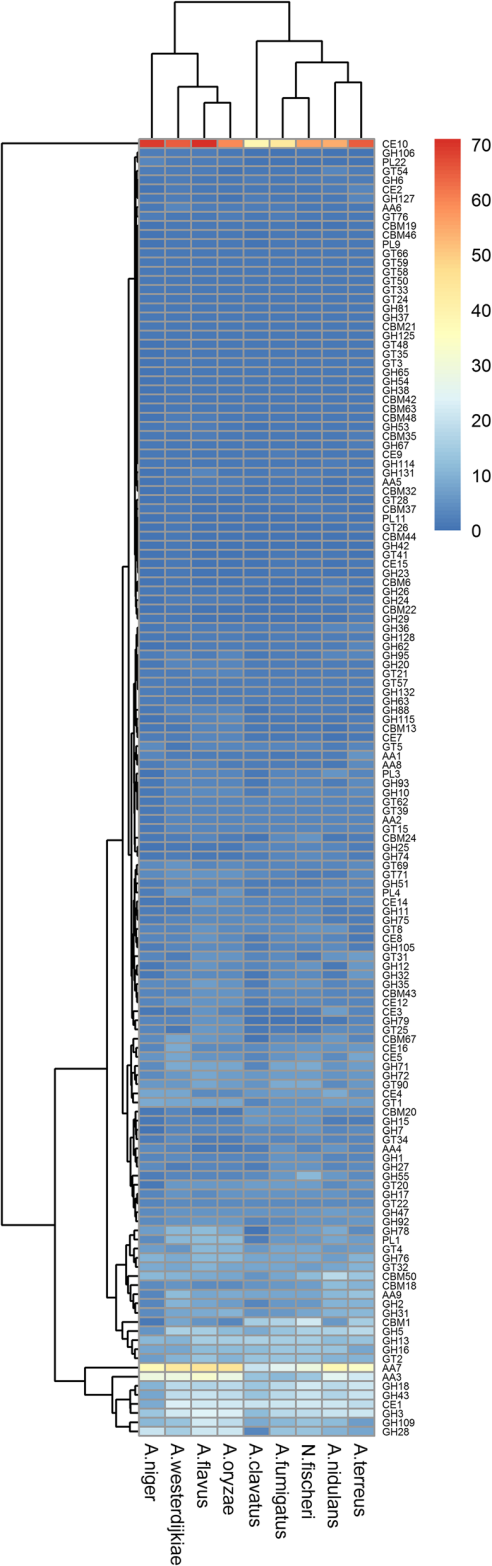
Comparison of the CAZymes identified in the genomes of the selected fungi using hierarchical clustering. The fungi analysed along with *A. westerdijkiae* were *A. oryzae, A. flavus, A. terreus, A. niger, A. fumigatus, N. fischeri, A. clavatus,* and *A. nidulans*. The enzyme families are represented by their classes (GH: glycoside hydrolases, GT: glycosyltransferases, PL: polysaccharide lyases, CE: carbohydrate esterases, and CBM: chitin binding modules) and the family numbers from the HMM predictions based on the carbohydrate-active enzyme database. The abundance levels of the different enzymes within a family are represented by a colour scale from the lowest (dark blue) to the highest occurrences (dark red) per species

Secreted CAZymes are crucial for fungal biological activity. Using the common CAZy annotation pipeline (see Methods section) for genomic analysis of fungi, which was also adopted to perform similar analyses in previous studies [39, 40], we explored the *A. westerdijkiae* genome for genes coding carbohydrate-active enzymes and carbohydrate-binding modules (CBMs). We identified 633 putative CAZy-coding genes falling into 142 families and 53 putative CBM-coding genes in 20 families (Additional file 5: Table S5). The Wilcoxon rank-sum test based on the gene counts in these families showed that the family distribution of the enzyme genes had no significant difference (*p* > 0.1) between *A. westerdijkiae* and the other *Aspergillus* genomes. The heatmap based on family classification suggested that *A. westerdijkiae,* along with *A. oryzae* and *A. flavus,* tend to be similar in degrading carbohydrates (Fig. 4). *A. niger*, *A. westerdijkiae*, *A. oryzae*, and *A. flavus* are known to colonize cereal grains, legumes, tree nuts, fruits, and vegetables, while the species in another clade, including *A. clavatus*, *A. fumigatus*, *N. fischeri*, *A. nidulans*, and *A. terreus*, are involved in decomposing vegetation and decaying organic matter, such as animal manure.

The glycoside hydrolase (GH), carbohydrate esterase (CE), and polysaccharide lyase (PL) groups include major plant polysaccharide degradation (PDD) enzyme families, also called cell wall-degrading enzymes (CWDE) due to their role in the disintegration of the plant cell wall exerted by bacterial and fungal pathogens [41]. The proteins containing CAZy domains in these families were considered as candidate proteins involved in the enzymatic degradation of plant polysaccharides (Additional file 5: Table S6). In the *A. westerdijkiae* genome, the percentages of PDD-related proteins in GH and PL groups were 60.5 and 91.7 %, respectively, which ranked together second only to *A. terreus* (60.7 and 93.3 %). We found that 23.4 % of the 633 putative CAZy-coding genes were PPD-related in the CE group, which had a low distribution in the *Aspergillus* genomes, which was comparable to the percentages found in other species [42].

Using comprehensive annotation and comparison analysis, we predicted 228 PDD-related enzymes, of which 187 (82 %) enzymes were annotated based on orthologous clustering. This annotation covered 180 PPD-related candidate proteins with CAZy domains (Additional file 4: Table S7). Of the remaining candidate proteins, 18 genes were inferred in terms of Kyoto Encyclopaedia of Genes and Genomes (KEGG), Conserved Domains Database (CDD), and Pfam annotations, whereas the 23 remaining potential PDD-related enzyme genes with only CAZy annotations were unknown due to the lack of reliable information, requiring further experimental data to infer their functions (Additional file 5: Table S8). With this information regarding the putative enzyme code, we compared the degradation potentials for cellulose, xyloglucan, xylan, galactomannan, pectin, starch, and insulin with those of the eight Aspergilli based on their genome content (Additional file 5: Table S9)[42]. We found that the predicted proteins of the *A. westerdijkiae* genome covered all of the enzyme activities, with many of them involved in pectin degradation.

In addition, the glycoside hydrolase family 18 (GH18), which contains all fungal chitinases, was responsible for the remodelling and recycling of the fungus’ own cell wall with other cell wall degrading enzymes [43]. GH18 was the third major family in the glycoside hydrolase class. Of 18 predicted enzymes in GH18, five were identified as secreted and pathogenic. The Auxiliary Activities (AAs) class contained enzymes with the potential capacity to help the original GH, PL, and CE enzymes gain access to the carbohydrates encrusted in the plant cell wall. In this class, the AA7 family contained 24 secreted enzymes. We also identified 12 secreted enzymes belonging to the family LPMO (AA9); these enzymes are crucial for lignin breakdown [44]. AAs contained the highest proportion (88.24 %) of the enzymes related to pathogenicity and virulence. CBM50 carbohydrate-binding modules, also known as LysM domains, were widely conserved in the fungal kingdom and might be functional in the virulence effects of plant pathogenic fungi for dampening host defence [45, 46]. Based on the CAZy annotation pipeline, we identified 10 predicted proteins containing CBM50 modules. In summary, 137 out of 274 putative secreted CAZymes were predicted to be potential PHI-related proteins. Most of these PHI-related CAZymes were in the family AA7 (23), GH3 (17), CE10 (16), AA3 (11), and GH28 (11) (Additional file 4: Table S4). These families may be observed in the close phylogenetic profiling clusters in Fig. 4.

### Proteases

Peptidases, which degrade proteins to provide an alternative carbon source, can be secreted during the infection process [47, 48]. Therefore, we performed a batch Blast search against the MEROPS protease database and identified 377 protease-coding genes, which were classified into 6 categories consisting of 91 subfamilies, and 7 inhibitor-coding genes (Additional file 6: Table S10). Of all of these putative proteases, 69 were predicted as secreted proteases, of which 39 exhibited homology with the pathogenicity- and virulence-related genes in the PHI database (Fig. 3) and belonged primarily to the families S09X (17), S10 (8), and A01A (8). All of the predicted proteases in the S09X family contained CE10 domains (Additional file 4: Table S4).

From a general view, the largest category of predicted proteases in the *A. westerdijkiae* genome was serine peptidases, with 176 genes belonging to 13 families. The top two families of predicted serine peptidases were prolyl oligopeptidase (79 genes) and prolyl aminopeptidase (58 genes). S9 was the largest family identified in the genome. Metallo (M) (103 genes) was the second largest protease category, of which glutamate carboxypeptidase (16 genes) was the largest family. Other abundant families were pepsin A (11 genes) within Aspartic (A) proteases, ubiquitin-specific peptidase 14 (17 genes) in Cysteine (C) proteases, and Archaean-proteasome beta component (14 genes) in Threonine (T) proteases.

### The biosynthetic potential of *A. westerdijkiae* suggested by a large number of secondary biosynthetic gene clusters

Filamentous fungi produce many bioactive secondary metabolites, such as various mycotoxins or other bioactive compounds that have been exploited in pharmaceuticals [49, 50]. The genes responsible for the production of the secondary metabolites tend to be organized in biosynthetic gene clusters [38]. Using the scaffolds as the query sequences of the antiSMASH 3.0 platform, we found many short and dense fragments present in some SM gene clusters, suggesting low prediction accuracy. Therefore, we constructed putative SM gene clusters according to the procedure described in the Methods section. In this case, a total of 88 putative secondary biosynthetic gene clusters, which spread on 21 scaffolds and a contig, were identified (Additional file 7: Table S11). The total length of these scaffolds and contig covered 89.96 % of the assembled sequences. Many of the putative SM gene clusters belong to type I *pks* (*t1pks*) or non-ribosomal peptide synthase (*nrps*) gene clusters, while the remainder were terpene, indole, or other hybrid gene clusters (Table 3). The number of putative *pks* gene, *nrps* gene and hybrid *nrps/pks* gene of *A. westerdijkiae* is comparable to other *Aspergilli* predicted by previous studies*,* such as *A. nomius, A. nidulans*, *A. flavus* and *A. oryzae* [51–53].

**Table 3:** Classification of putative secondary metabolite clusters of *A. westerdijkiae* predicted by antiSMASH

Type	Count
T1pks	25
NRPS	17
NRPS-T1PKS hybrid	8
Terpene	8
Terpene-T1PKS hybrid	2
Indole	6
Indole-NRPS hybrid	3
Siderophore	1
Other	18
Total	88

**Fig. 5:**
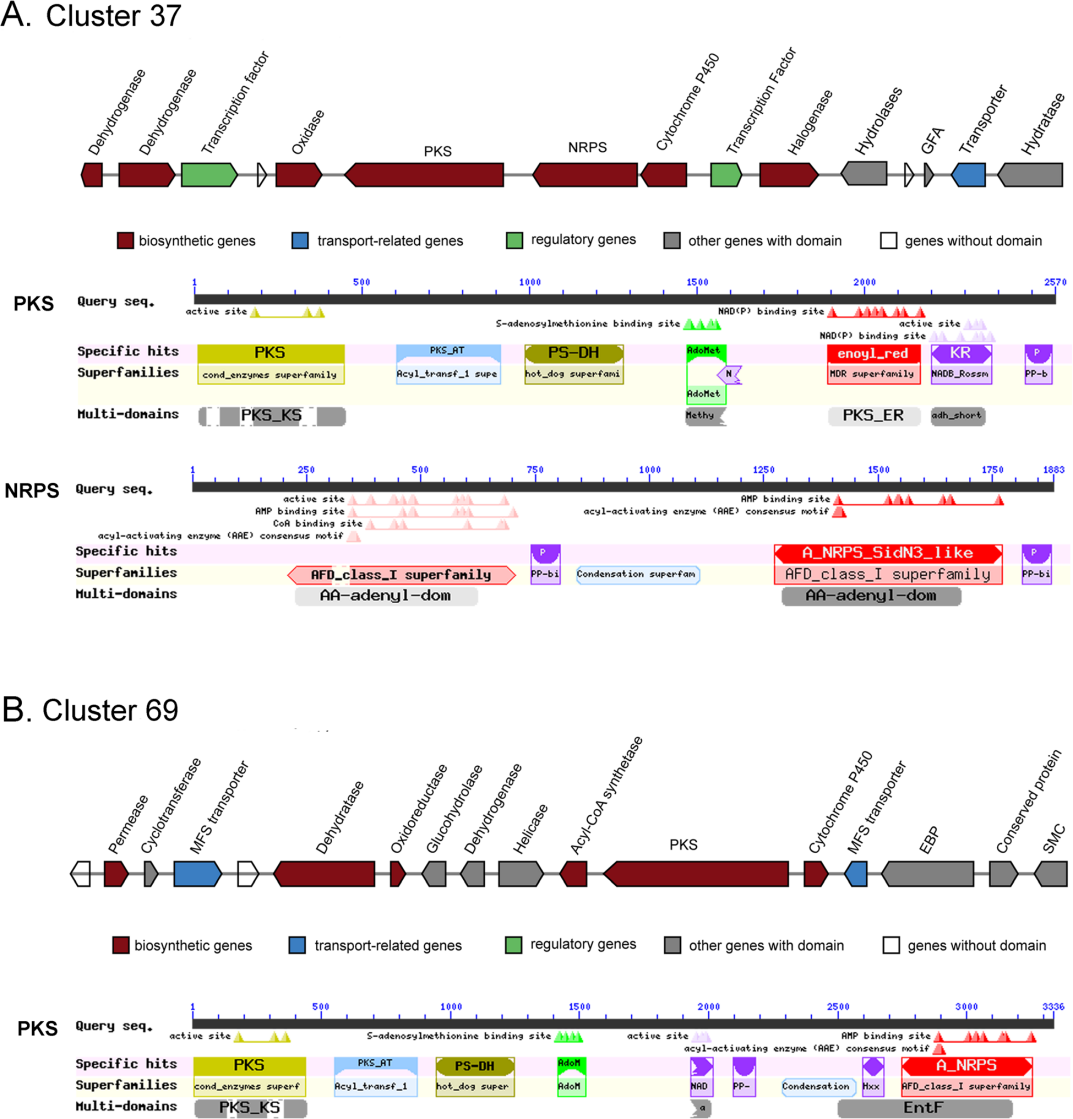
Two OTA biosynthesis-related gene clusters for *A. westerdijkiae*. **a** Cluster37, with 15 genes located on scaffold14 (399784-445042 nt). **b** Cluster69, with 17 genes located on scaffold45 (263918-334751 nt). The *PKS* and *NRPS* domains were determined by the Blast searches against the CDD database

The previous studies successively concluded two portions of the sequences of the *t1pks* genes involved in the biosynthesis of the OTA mycotoxin [15, 16]. Silencing either of the two genes might inhibit OTA production, suggesting that the two clusters jointly play a role [2]. In this study, we predicted more complete sequences of the two *t1pks* genes and classified them into two distinct putative SM gene clusters: i) Cluster37, located on scaffold14 (locus: 390369–4449037), includes 15 genes; and ii) Cluster69, located on scaffold45 (locus: 255712–339653), includes 17 genes(Fig. 5, Additional file 7). The genes in cluster37 successively encoded a *pks* (awe04182), an nrps (awe04183), a cytochrome P450 monooxygenase (awe04184), a bZIP transcription regulator (awe04185), and a halogenase (awe04186) (Fig. 5a, Additional file 8: Table S12). They shared 66.67, 56.62, 67.32, 42.86, and 77.14 % amino acid identity with 5 out of 6 co-expressed genes (CEGs) (173482, 132610, 517149, 7821 and 209543) in cluster38 of *Aspergillus carbonarius*, respectively [54]. However, the rest of the genes in both clusters were non-homologous under the Blast search E-value threshold of 1e-10. In addition, cluster37 of *A. westerdijkiae* also included a Zn2Cys transcription regulator (awe04179) and a sugar transporter (awe04190).

The putative cluster69 encoded a *pks* (sc45_org87), a cytochrome P450 monooxygenase (awe08996), an Acyl-CoA synthetase (awe08993), and two transporters (awe08996, awe08997) (Fig. 5b, Additional file 8: Table S13). The *pks* gene was originally classified as two separate genes (awe08994, awe08995) by the MAKE2 prediction pipeline. Further domain prediction and homology analysis suggested the two genes should be integrated despite the junction showing low similarity in Blast search. Therefore, we chose the gene model sc45_org87 predicted by antiSMASH as the integrated gene in this cluster, which shares 32.42 % amino acid identity with the CEG 511653 in cluster4 of *Aspergillus carbonarius*, while awe08996 shares 31.22 % amino acid identity with the adjacent CEG 392816 coding a cytochrome P450 monooxygenase. No additional homologous genes were found between these two clusters. A putative acetyl-CoA synthetase was predicted to be present next to the *pks*. Acetyl-CoA, used as a carbon source in polyketide biosynthesis, is likely to be a precursor for OTA synthesis [15, 55]. We subsequently identified a GH3 enzyme gene, acting as a beta glucosidase, in the 3’ direction of the *pks* gene. Pathway analysis indicated that this putative GH3 enzyme gene participated in starch and sucrose metabolism. Based on the Blast and domain analyses, we also predicted an AA3 enzyme gene close to the GH3 enzyme genes. The predicted AA3 enzyme gene belonged to the glucose-methanol-choline (GMC) oxidoreductase family and could be further categorized into the AA3_2 family containing glucose 1-oxidase. We found that the GH3 and AA3 enzymes could be classified into the close phylogenetic profiling clusters summarized in the heatmap of CAZymes, which suggested that the AA3 enzymes might act in conjunction with GH3 enzymes. Moreover, it was observed that these two genes did not belong to any orthologous cluster. In summary, these two genes might have important influences on OTA production in various substrates. This suggestion is in agreement with the observation that *A. westerdijkiae* possessed the highest capability for producing OTA in media containing high amounts of sucrose and glucose, such as paprika-based medium, while OTA was absent in grape-based medium, in which fructose is the most abundant compound [24]. With domain prediction, the structure of *pks* in putative cluster37 *of A. westerdijkiae*, the partial sequence of which was reported as LC35-12 [GenBank: AAT92023.1), was KS-AT-DH-MT-ER-KR-ACP [16, 56]. The structure of *pks* in putative cluster69 *of A. westerdijkiae*, which shows high identity (649/670) with the validated OTA-related portion of “*aoks1”* [GenBank: AAT92024.1], was KS-AT-DH-MT-KR-ACP-C-A. These two putative structures of *pks* could be divided into two different fungus-reducing *PKS* clades: the former was clade I, while the latter was clade II due to the loss of the ER domain [57]. We also detected a putative methylsalicylic acid *pks* gene (awe07918) with 96.22 % sequence identity with “*aomsas*” [GenBank: AAS98200.1], *pks* gene involved in the biosynthesis of isoasperlactone and asperlactone and reported previously in *A. westerdijkiae* [58].

**Fig. 6:**
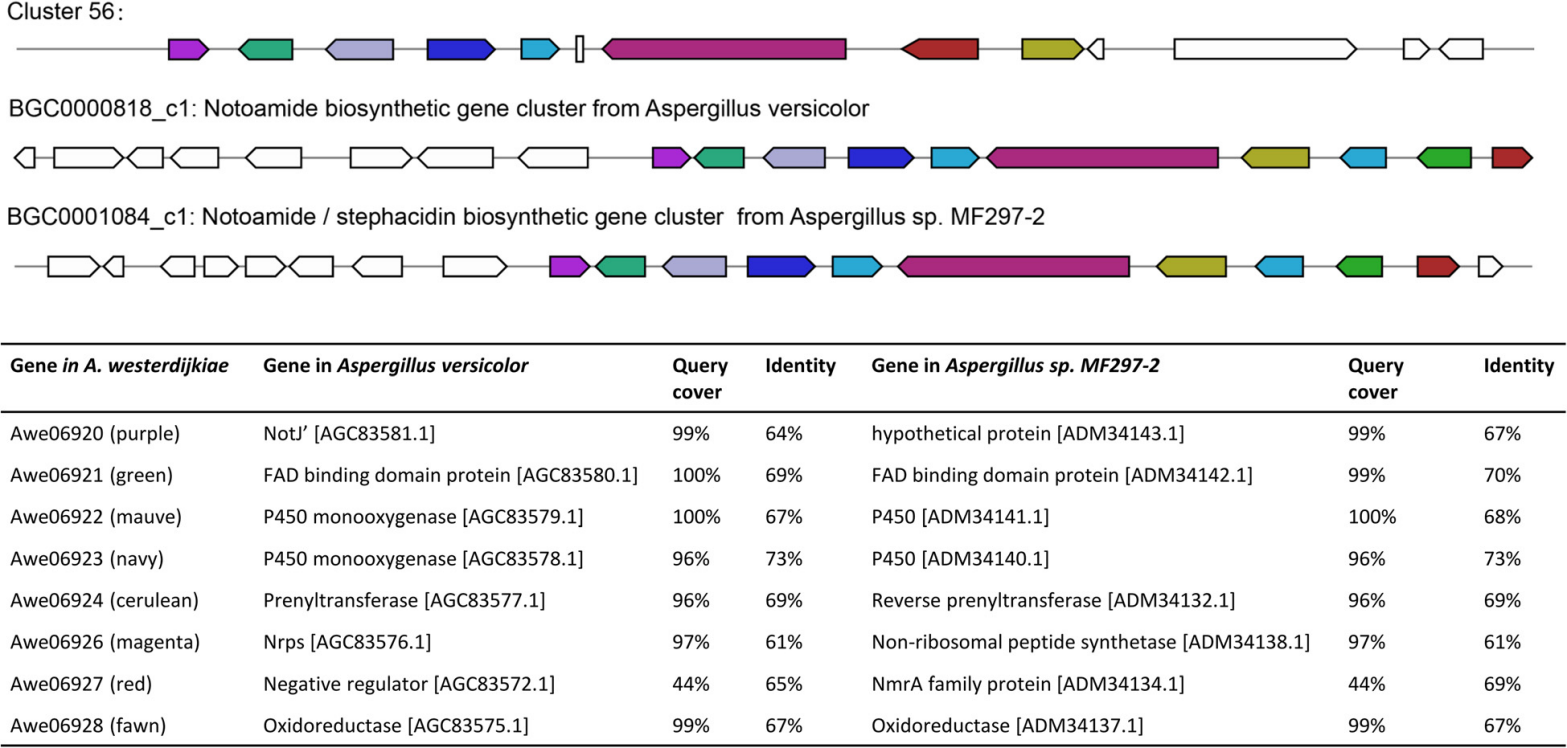
The putative notoamide biosynthetic gene cluster of *A. westerdijkiae* found in this study and the comparison of this cluster with the notoamide cluster reported for *Aspergillus versicolor* and *Aspergillus sp. MF297-2.* Gene cluster description of *Aspergillus versicolor* and *Aspergillus sp. MF297-2* was acquired from Minimum Information about a Biosynthetic Gene cluster (MIBiG) database under accession number BGC0000818 (GenBank: JQ708194, positions: 1–43815) and BGC0001084 (GenBank: HM622670, positions: 1–42456), respectively

**Fig. 7:**
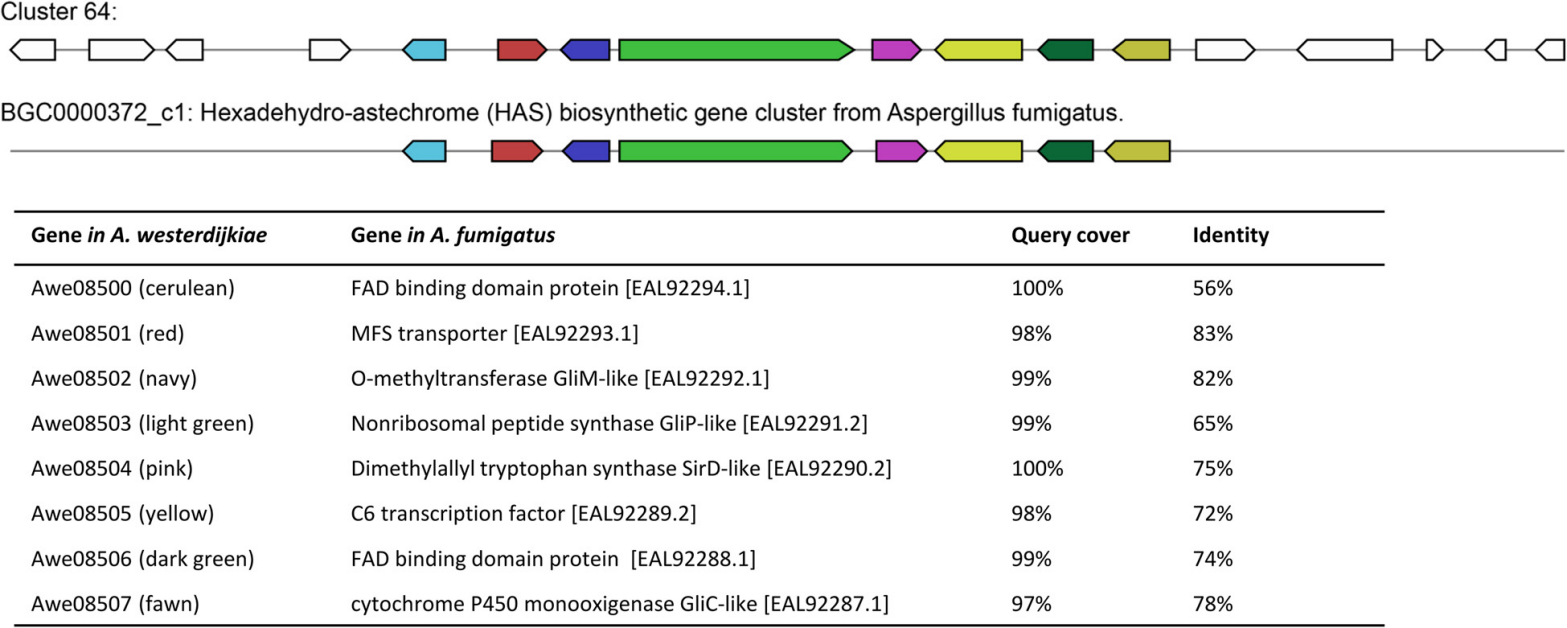
The putative hexadehydro-astechrome (HAS) biosynthetic gene cluster of *A. westergijkiae* found in this study and the comparison of this cluster with the hexadehydro-astechrome cluster reported for *Aspergillus fumigatus Af239*. Gene cluster description of *A. fumigatus* was acquired from MIBiG database under accession number BGC0000372 (GenBank CM000171, positions 3423866–3446129)

Non-ribosomal peptide synthase *genes* are responsible for the synthesis of natural peptide-based microbial products. We identified a total of 17 putative *nrps genes* distributed in different gene clusters in the *A. westerdijkiae* genome. We further predicted a notoamide biosynthetic gene cluster centred on an *nrps* gene in the *A. westerdijkiae* genome, suggesting that *A. westerdijkiae* should be able to produce notoamide and the related structural derivatives (Fig. 6) [59, 60]. Moreover, because of the homology shared with the characterized biosynthetic gene clusters in *A. fumigatus Af239*, *A. westerdijkiae* is likely to produce the widely occurring siderophore hexadehydro-astechrome (HAS) (Fig. 7) [61–64]. In the *A. westerdijkiae* genome, we also predicted several *nrps* gene clusters producing di- and three-peptide alkaloids containing the amino acid anthranilate.

In *A. westerdijkiae,* we also predicted several hybrid *nrps/pks* gene whose products remain to be determined. In addition to the *pks*, *nrps*, and hybrid *nrps/pks* gene clusters, we also identified 10 gene clusters likely to produce *terpenes*, including two hybrid *terpene-pks* gene clusters. In this study, we were able to observe almost all of the secondary metabolism clusters containing the reported genes or gene portions.

## Conclusions

This genome-wide comparative study facilitated our understanding of the evolutionary relationships between *A. westerdijkiae* and other sequenced *Aspergillus* species. The analysis of genome characteristics and evolutionary relationships provided evidence suggesting that the *A. westerdijkiae* genome was most closely related to the *A. oryzae genome. A. westerdijkiae* was capable of producing abundant plant polysaccharide-degrading enzymes and secreted proteases, and the results related to these findings in our study might serve as a basis for understanding the principles of *A. westerdijkiae* colonization and pathogenicity. With respect to the secondary metabolites, we identified numerous secondary biosynthetic gene clusters, including OTA gene clusters and a few other clusters with predicted products. Some of these gene clusters did not share similarity with any characterized biosynthetic gene clusters, indicating that *A. westerdijkiae* could potentially produce novel secondary metabolites. These findings set the stage for later experimental studies and might be helpful for further understanding the pathogenicity of *A. westerdijkiae*.

## Methods

### Culturing and extraction of genomic DNA

*Aspergillus westerdijkiae* CBS112803 strain was obtained from Centraalbureau Voor Schimmelculture, Netherlands (CBS) and was cultured in 50-ml flasks containing LMM broth for 6 days at 25 °C with shaking at 120 rpm. The fungal mycelial mat was harvested and ground into a fine powder with liquid nitrogen in a mortar. The concentration of the DNA sample was measured using a NanoDrop spectrophotometer, and the sample was resolved on an agarose gel before it was sent to Macrogen Inc., Korea for whole-genome sequencing.

### Genome sequencing and assembly

For the whole-genome shotgun sequencing of *A. westerdijkiae*, the Illumina MiSeq platform was adopted with two meta-pair libraries, one 3 kb and the other 10 kb [29]. The raw sequence data coming from the high-throughput sequencing pipelines were applied to the program FastQC (http://www.bioinformatics.babraham.ac.uk/projects/fastqc) for quality control of sequencing. The reads were filtered before assembly such that for a pair of paired-end (PE) reads, each read should have more than 90 % of bases with a base quality greater than or equal to Q20. The contigs and scaffolds were assembled using the short-read assembly tool SOAPdenovo2 [65]. CEGMA, a bioinformatics tool for assessing the completeness of the gene space, was employed with a refined set of 248 core eukaryotic genes to evaluate the assembly efficiency of the sequenced genome [30]. To identify genome repetitive sequences, assembled scaffolds were supplied to RepeatMasker (RMLib: 20140131 & Dfam: 1.3 as fungi repetitive sequence library) [66].

### Genome annotation and classification

After masking the identified repetitive sequences, gene prediction in *A. westerdijkiae* was implemented according to the MAKER2 pipeline [67, 68]. An initial run of MAKER v2.31.8 was performed to construct the phylogenetic tree using ab initio gene-predictor SNAP v2013-11-29 [69]. To train the SNAP, CEGMA, a bioinformatics tool for building a highly reliable set of gene annotations in the absence of experimental data, was adopted [70]. All expressed sequence tags (ESTs) and protein sequences in the Refseq (75,695), UniProtKB/Swiss-Prot (3617), and GenBank (148,141) databases via the NCBI Taxonomy (by February 2015) (http://www.ncbi.nlm.nih.gov/taxonomy) were pooled as alternative EST and protein homology evidence. A subsequent run of MAKER was performed to increase the sensitivity of gene identification. AUGUSTUS v2.55 [71] was added into the initial MAKER pipeline. According to the evolutionary relationships described above, we selected “*Aspergillus oryzae*” as the default training set of AUGUSTUS. The final outcome was reached by combining the predictions of SNAP and AUGUSTUS. The putative proteins were aligned against the NCBI *nr*, UniProtKB (Swiss-Prot and TrEMBL), and KEGG databases using BLASTP with the cutoff E-value set at 1e-10. The predicted genes were then aligned against the CDD database using rpsBLAST. The domain compositions were analysed by performing a HMMER v3.1b1 (http://hmmer.org/) scan based on the profiles compiled from Pfam (by February 2015).

All proteomes were scanned to predict subcellular localization using TargetP v1.1b [72]. The remaining proteins were investigated using the Hidden Markov Model (HMM) in SignalP v4.1 to look for signal peptides [73]. These proteins were then scanned for the presence of transmembrane domains using TMHMM v2.0c [74]. To reduce redundancy, the secreted proteins were clustered using CD-HIT (v4.5.4, with default parameters) if they shared more than 90 % identity over a range of above 50 % of the sequence length [75]. A single representative sequence was selected from each protein cluster. To evaluate the potential of a gene to produce secondary metabolites, both the assembled scaffolds and the 10,861 predicted proteins of the *A. westerdijkiae* genome were supplied to antiSMASH (v3.0.5, with default parameters, except for checking the “DNA of Eukaryotic Origin” box) [76]. To conclude the structure of each predicted SM cluster, the following rules were adopted: firstly, the boundaries were determined in accordance with the outputs from antiSMASH, with the scaffolds as the query submission; secondly, the predicted proteins, which were located in the intervals or overlapped with each side of the boundaries, were selected to construct the final clusters; lastly, the SM gene clusters of interest were further examined according to the domain annotations. To identify genes involved in pathogenicity and virulence in the *A. westerdijkiae* genome, Blastp with a cut-off E-value set at 1e-10 was adopted to search against the Pathogen Host Interactions (PHI) database (by April 2015), which contains experimentally validated pathogenicity, virulence, and effector genes of fungal, oomycete, and bacterial pathogens [77]. The genome-encoding cytochrome P450s were annotated using Blastp to search the fungal Cytochrome P450 database (by April 2015) with a cut-off E-value set at 1e-10 [78, 79]. Proteomes were classified into proteolytic enzyme families by performing a batch Blast search against the MEROPS protease database (release 9.13) [80, 81], and carbohydrate-active enzymes were classified using a HMMER (v3.1b1, with default parameters) scan against the profiles compiled with dbCAN release 4.0 [82] based on the CAZy database [83]. Based on orthologue analysis and functional annotation, PDD enzyme-related genes were screened and classified into different enzyme coding categories [22, 42]. Statistical comparisons of carbohydrate active enzymes and peptidases between *A. westerdijkiae* and the eight other *Aspergillus* species were made using the Wilcoxon rank-sum test in the R platform.

### Genome comparative analyses

Pair-wise sequence alignments between *A. westerdijkiae* and the eight other sequenced Aspergillus species, of which four genome sequences have been published (*A. fumigatus* [84], *A. nidulans* [32], *A. niger* [85], *A. oryzae* [34]) and four others assembled and annotated (*A. flavus* [86], *A. clavatus* [36], *A. terreus* [36], *Neosartorya fischeri* [36]), were performed using the *Nucmer* and *Promer* programs in the MUMmer v3.23 package (http://mummer.sourceforge.net/) [31]. The corresponding chromosome information was acquired from the Aspergillus genome database (AspGD) (May 2015) (http://www.aspergillusgenome.org/). Protein sequences of the other eight species presented in the comparative analysis were all acquired from the Aspergillus Comparative Database (May 2015) (http://www.broadinstitute.org/). From the eight *Aspergillus* species, supercontigs larger than 100 kb were aligned against the 29 largest *A. westerdijkiae* supercontigs.

The phylogenetic tree of *A. westerdijkiae* and the other eight *Aspergillus* species was constructed using whole genome-wide sequences. Orthologous protein prediction was performed using Proteinortho (v5.11, with default parameters, except that identity = 75) [87]. Among the predicted orthologous gene clusters, 561 highly conserved single-copy gene clusters were chosen and aligned using MUSCLE (v3.8.31_i86linux64, with default parameters) [88]. To remove divergence and ambiguously aligned blocks from the alignment, Gblocks (v0.91b) [89] was employed under the default parameter setting. Trimmed alignments of orthologous sequences were concatenated using a Perl script FASconCAT (v1.02, with default parameters) [90], and a maximum likelihood phylogenetic tree was created using the Dayhoff model in TREE-PUZZLE v5.3.rc16 [91] with 1000 bootstrap replicates. The tree was visualized using Figtree v1.42 (http://tree.bio.ed.ac.uk/software/figtree).

## Abbreviations

antiSMASH, Antibiotics & Secondary Metabolite Analysis Shell; AspGD, Aspergillus genome database; BLAST, Basic local alignment search tool; CAZyme, Carbohydrate activity enzyme; CBM, Carbohydrate binding module; CDD, Conserved Domains Database; CE, Carbohydrate esterase; CEG, Co-expressed gene; CWDE, Cell wall degrading enzyme; CYP450, Cytochrome P450; GH, Glycoside hydrolases; GT, Glycosyl transferase; HAS, Hexadehydro-astechrome; KEGG, Kyoto Encyclopaedia of Genes and Genomes; ML, Maximum likelihood; nr, NCBI non-redundant; NRPS, Non-ribosomal peptide synthase; OTA, Ochratoxin A; PHI, Pathogen-host interaction; PKS, Polyketide synthase; PL, Polysaccharide lyase; PPD, Plant polysaccharide degradation; SM, Secondary metabolite; T1PKS, Type I PKS

## Additional files

Additional file 1: Table S1. Statistics of the Aspergillus genomes used in this study. **Table S2**. Scaffolds greater than 100 kb in the A. westerdijkiae genome sequences. (XLSX 13 kb) Additional file 2: Sequences and coordinates of the predicted proteins and transcripts of *A. westerdijkiae*. (ZIP 10770 kb) Additional file 3: Table S3. Genome-wide alignment resulting from the MUMmer comparisons. (DOCX 21 kb) Additional file 4: Table S4. Putative secreted proteins involved in PHI, CAZy, Protease, and CYP450. (XLSX 134 kb) Additional file 5: Table S5 Counts of the putative genes per CAZy family in the 9 genomes used in this study. **Table S6**. Counts of the putative genes per Plant Polysaccharide Degradation (PDD)-related CAZy family in the 9 genomes used in this study. **Table S7**. Functional annotation of the PDD-related genes (with orthologues) for *A. westerdijkiae*. **Table S8**. Functional annotation of the PDD-related genes (without orthologues) for *A. westerdijkiae*. **Table S9**. Counts of the putative genes involved in plant polysaccharide degradation in the nine Aspergillus species. (XLSX 79 kb) Additional file 6: Table S10. Putative proteases predicted by the batch Blast against the MEROPS database. (XLSX 53 kb) Additional file 7: Table S11. Putative secondary metabolism clusters. (XLSX 18 kb) Additional file 8: Table S12. Structural and functional annotations of the OTA biosynthesis-related clusters on scaffold14 of *A. westerdijkiae*. **Table S13**. Structural and functional annotations of the OTA biosynthesis-related cluster on scaffold45 of *A. westerdijkiae*. (DOCX 153 kb)
