# Supplementary material for: Sequencing and functional annotation of the whole genome of the filamentous fungus Aspergillus westerdijkiae
Source: BMC Genomics. 2016 Aug 15;17:633. doi: 10.1186/s12864-016-2974-x (PMC4986183; doi:10.1186/s12864-016-2974-x)
Supplement: Additional file 3: Table S3. — Genome-wide alignment resulting from the MUMmer comparisons. (DOCX 21 kb) [file 12864_2016_2974_MOESM3_ESM.docx]

**Table S3. Genome-wide alignment resulting from the MUMmer comparisons.**

| Query | Reference | Nucmer | | Promer | |
| --- | --- | --- | --- | --- | --- |
|  |  | **Aligned length (bp)** | **Average identity (%)** | **Aligned length (bp)** | **Average identity (%)** |
| *A. clavatus* | *A. westerdijkiae* | 487706 | 89.2544 | 21832218 | 67.8614 |
| *N. fischeri* | *A. westerdijkiae* | 472928 | 88.3747 | 23436345 | 67.964 |
| *A. flavus* | *A. westerdijkiae* | 533522 | 87.2899 | 26633775 | 68.2262 |
| *A. fumigatus* | *A. westerdijkiae* | 366138 | 87.9771 | 22049151 | 68.1892 |
| *A. nidulans* | *A. westerdijkiae* | 217347 | 87.021 | 19634388 | 67.2677 |
| *A. niger* | *A. westerdijkiae* | 633782 | 87.3753 | 25004649 | 67.8927 |
| *A. oryzae* | *A. westerdijkiae* | 545226 | 87.8822 | 27055911 | 68.3502 |
| *A. terreus* | *A. westerdijkiae* | 699991 | 86.6345 | 25533699 | 67.8072 |
