# Supplementary material for: Sequencing and functional annotation of the whole genome of the filamentous fungus Aspergillus westerdijkiae
Source: BMC Genomics. 2016 Aug 15;17:633. doi: 10.1186/s12864-016-2974-x (PMC4986183; doi:10.1186/s12864-016-2974-x)
Supplement: Additional file 8: Table S12. — Structural and functional annotations of the OTA biosynthesis-related clusters on scaffold14 of A. westerdijkiae. Table S13. Structural and functional annotations of the OTA biosynthesis-related cluster on scaffold45 of A. westerdijkiae. (DOCX 153 kb) [file 12864_2016_2974_MOESM8_ESM.docx]

**Table S12. Structural and functional annotations of the OTA biosynthesis-related clusters on scaffold14 of A. westerdijkiae.**

| 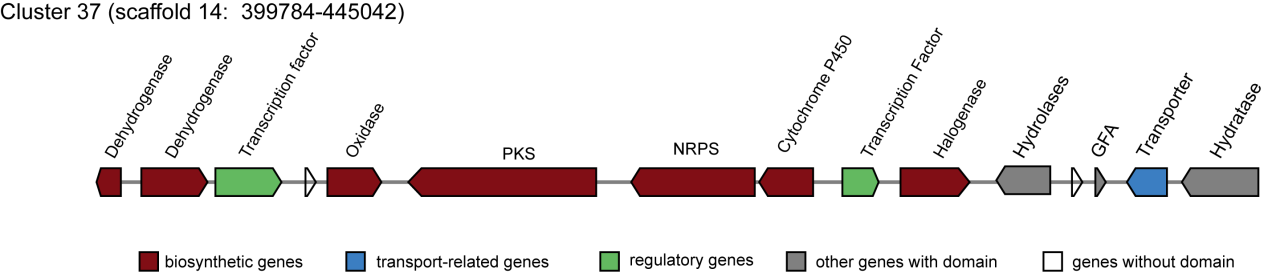 | | | | |
| --- | --- | --- | --- | --- |
| No | **Name** | **Location** | **Function / domain description** | **KEGG Pathway** |
| 1 | Awe04177 | 399367-400379 (-) | Mannitol dehydrogenase (MDH)-like, classical (c) SDRs | ko00061, Fatty acid biosynthesis;  ko00780, Biotin metabolism;  ko01040, Biosynthesis of unsaturated fatty acids |
| 2 | Awe04178 | 400882-404572 (+) | Sorbitol dehydrogenase | ko00051, Fructose and mannose metabolism |
| 3 | Awe04179 | 404831-406967 (+) | GAL4-like Zn2Cys6 binuclear cluster DNA-binding;  Fungal transcription factor regulator |  |
| 4 | Awe04180 | 407512-407826 (+) | Unknown |  |
| 5 | Awe04181 | 408624-409990 (+) | Glycine/D-amino acid oxidases (deaminating) [Amino acid transport and metabolism] |  |
| 6 | Awe04182 | 410767-418909 (-) | PKS [KS-AT-DH-MT-ER-KR-ACP] |  |
| 7 | Awe04183 | 419784-425484 (-) | NRPS [A-PP-C-A-PP] | ko01054, Nonribosomal peptide structures |
| 8 | Awe04184 | 426287-428113 (-) | Cytochrome P450 |  |
| 9 | Awe04185 | 429271-430037 (+) | Basic leucine zipper (bZIP) domain of Activating Transcription Factor-2 (ATF-2) |  |
| 10 | Awe04186 | 433195-434970 (+) | Tryptophan halogenase |  |
| 11 | Awe04187 | 436415-438133 (-) | Peptidase S33 tripeptidyl aminopeptidase-like |  |
| 12 | Awe04188 | 439532-439969 (+) | Unknown |  |
| 13 | Awe04189 | 441094-441662 (+) | Glutathione-dependent formaldehyde-activating enzyme |  |
| 14 | Awe04190 | 442845-444738 (-) | Sugar transportor | ko04113, Meiosis - yeast |
| 15 | Awe04191 | 445446-447905 (-) | Aconitate hydratase | ko00020, Citrate cycle (TCA cycle);  ko00630, Glyoxylate and dicarboxylate metabolism |

**Table S13. Structural and functional annotations of the OTA biosynthesis-related cluster on scaffold45 of A. westerdijkiae.**

| 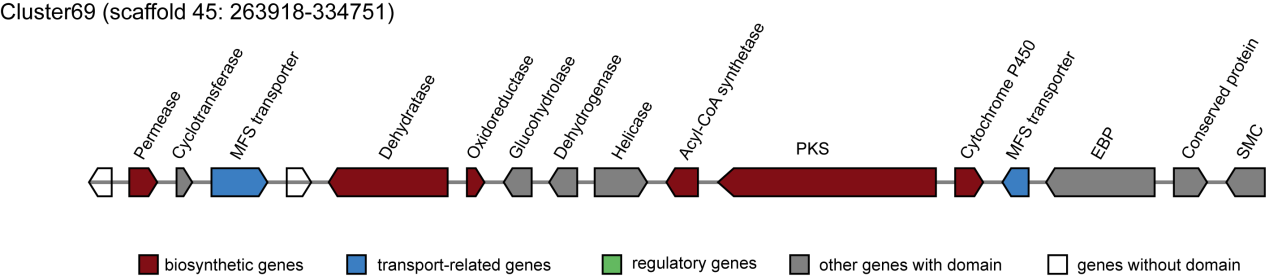 | | | | |
| --- | --- | --- | --- | --- |
| No | **Gene** | **Location** | **Function / domain description** | **KEGG Pathway** |
| 1 | Awe08983 | 265664-266965 (-) | Conserved hypothetical protein without domain |  |
| 2 | Awe08984 | 270899-272703 (+) | Amino acid permease (GABA permease) |  |
| 3 | Awe08985 | 273459-274319 (+) | Gamma-glutamyl cyclotransferase (GGCT) |  |
| 4 | Awe08986 | 274955-279050 (+) | MFS transporter, ACS family, allantoate permease |  |
| 5 | Awe08987 | 279727-281082 (+) | Hypothetical protein without domain |  |
| 6 | Awe08988 | 281762-289659 (-) | D-galactonate dehydratase | ko00052, Galactose metabolism |
| 7 | Awe08989 | 290248-291616 (+) | Aldo-keto reductase family oxidoreductase (related to aryl-alcohol dehydrogenase) | ko00010, Glycolysis / Gluconeogenesis;  ko00561, Glycerolipid metabolism;  ko00930, Caprolactam degradation |
| 8 | Awe08990 | 292104-294548 (-) | Beta-D-glucoside glucohydrolase (GH3) | ko00460, Cyanoamino acid metabolism;  ko00500, Starch and sucrose metabolism;  ko00940, Phenylpropanoid biosynthesis; |
| 9 | Awe08991 | 295600-297168 (-) | glucose-methanol-choline (GMC) oxidoreductases (AA3_2) | ko00260, Glycine, serine and threonine metabolism; |
| 10 | Awe08992 | 298139-301082 (+) | Superfamily II DNA or RNA helicase, SNF2 family [Transcription, Replication, recombination and repair] | ko03440, Homologous recombination |
| 11 | Awe08993 | 301377-303326 (-) | Acyl-CoA synthetase (AMP-forming)/AMP-acid ligase II [Lipid transport and metabolism, Secondary metabolites biosynthesis, transport and catabolism] |  |
| 12 | Sc45_org87* | 303660-314968 (-) | PKS [KS-AT-DH-MT-KR-ACP-C-A] |  |
| 13 | Awe08996 | 315177-316778 (+) | Cytochrome P450, family 94, subfamily C protein; | ko00363, Bisphenol degradation;  ko00624, Polycyclic aromatic hydrocarbon degradation;  ko00627, Aminobenzoate degradation;  ko00903, Limonene and pinene degradation;  ko00945, Stilbenoid, diarylheptanoid and gingerol biosynthesis |
| 14 | Awe08997 | 317568-319202 (-) | Major Facilitator Superfamily; Monocarboxylate transporter 1 |  |
| 15 | Awe08998 | 320329-327671 (-) | Emopamil binding protein (EBP) | ko00100, Steroid biosynthesis |
| 16 | Awe08999 | 328280-330445 (+) | Uncharacterized conserved protein (DUF4484 superfamily) |  |
| 17 | Awe09000 | 331140-333281 (-) | Chromosome segregation protein (SMC) |  |

* >Sc45_org87 | scaffold 45: 303660-314968 (-)

MDYGDKPIAIIGIGCRFPGSSSTPAKFWDLLSKPKHVGSPVPKDRFDGDAFYNTRSANHGTTNASESYFLRENVAAFDASFFNISAREAESIDPQQRVLLETVYEAVEAAGLRLEDLRGSPTGIFCGVMCDDYQTIQQRDVSELPHYTATGTARSIISNRVSYFFDWHGPSMTIDTACSSSLVALHLAAKALHDGECRVVVASGTNLILAPNMYISESKLSMLSPHGRSRMWDAGADGYARGEGVAAVALKRLSDAIADGDAIECIIPATHINQDGRSMGITMPSSTAQTDLIRSTYRKAGLDPANPRDRCQYFEAHGTGTPAGDPQEASAIHAAFFPNPVDDKSDPDNRLFVGSAKTVVGHTEGTAGLAALIKGVMSLQHGVVAPNLHFERLNPAIEPYTRQLRVPTDCQPWPQLPEGTPRRVSVNSFGFGGTNAHAILEAYSPTLSDNNEWDGIIPFVFTAPSDKALGNVLTAYVDYLDGQQPTANLGDLAWTLYRRRTTFSHRVALWAASARDLQNRLREEVARRGSNQASTVISKPRTDRPKVLGVFTGQGAQWAQMGLDLVQRSPDAEKWLTTLQMALDGLPDEYRPAYSILDELCAESEKSRLHLAEISQPLCTAVQIVLVKFLRSLGIDLNAVVGHSSGEIAAAYAAGIVSEIEAIRIAHLRGHVTSLAGCNGQPGSMLAVGMSPDEADQVCQSAVYTGRIKPAAVNSSSSITLSGDADAIVALEAQLKDEGIFARRLKVNMAYHSHQMIPCSAPYLRALESCQIQPREPKDTKWFSSVNDGRIVDATHLEVLCGSYWCDNMVQTVRFADAVTQALQDDSYDMIVEVGPHPALKSPVLGTLSETSLAQGTAQVPPVYTSLLNRSTSGTECVARAIGDIYTHLGPDAVDVESYMRHFRDRPTFHLAKGLPCYPFDRSNSYWAESRFSRATFRQAGRPNQLLGSSCAGTTDTAYRWRNFLHTSEIDWLSGHRIQSQTVFPATGYVAMALEAAYV...CYHLDNEGPYQAILASSPLCLNVARVGSEAEVRDLYLGLRQHVFDLESGDNIRMNLAITPKNQQFLLIAFHHICLDGLSFQLLVGELERAYMQQPLSPMARQYADYAAAQRASYEAGHVSKDVAYWRQEFSTFPSPIPLFPMARVPARTTLTDHPRENVRIELPASIMETVQSLGKCMRITPFGIFLAVFRVFLARLTQSTDFCIGISDVHRIEEADERLIGLVQNLLPLRFVGSLEGRSFREVLLDTQTKARGALAHSRVQFDRLLDELAAPRSGSHSPLFQVMLDWQPSSAEKRWFAGLEIDVQEWAINKTAFDMVLSVMDSGQGTSVLNFHLQQALFTRDAAHLIARSFISLLEDLVSSDPVQVVTGPSLYPRTDIDSALALGRGPEMPSQWQPTLSRRIAEIARVSSDQMAVIDPVSGRGLTYAALMHRSSAVASHLAELVVGPASTVCLFQQPTESWIVCMLAIWRLGAVYVPLDVNSPRDRLAIVIEDCQPQVIICDDDTESALHTMAVASHSSVVNTSAVDMNGKPTTRETADSSSAHASAVILYSSGTTGRPKGFQLSHANLQNQLEGFTRQCGLQAPVVLQQGATTFDISLEQALTGLTTGGQVVIAPRSVRGDPAALARIIVDQRITCTMATPSEYLLWMQHASETLKTASDTWTMAFSGGEAFPGSLPAVFADLQLDHLRLINFYGPGETTIASHQIEVDYRRQDGGSFEGTVVPVGHALPNYTTYIVDTEGNPVPTGISGEIVIGGAGPCLGYLHLDTLTQTQFVHDRHATPANNAQGWTRAYRTSEKGHMLQNGALVLEGRLDGDSQVKLRGIRMDLGDIENAILGTARGALNRVVATLRDAADGSSFLVAHVEFVPESTICDKQVFLRHVRAALPLPQNMRPSLIVPVESMPTTAHGKLDRRVIKELPLPERKRSSLMVDPSVLATTTDWVGRVLLAWRFDAKIPLIDLVEGSTLGEMAEAARSAATGRSSVAIPMETRALELS
